# Evidential Value That Exercise Improves BMI *z*-Score in Overweight and Obese Children and Adolescents

**DOI:** 10.1155/2015/151985

**Published:** 2015-10-05

**Authors:** George A. Kelley, Kristi S. Kelley

**Affiliations:** Department of Biostatistics, West Virginia University, Morgantown, WV 26506-9190, USA

## Abstract

*Background.* Given the cardiovascular disease (CVD) related importance of understanding the true effects of exercise on adiposity in overweight and obese children and adolescents, this study examined whether there is evidential value to rule out excessive and inappropriate reporting of statistically significant results, a major problem in the published literature, with respect to exercise-induced improvements in BMI *z*-score among overweight and obese children and adolescents. *Methods.* Using data from a previous meta-analysis of 10 published studies that included 835 overweight and obese children and adolescents, a novel, recently developed approach (*p*-curve) was used to test for evidential value and rule out selective reporting of findings. Chi-squared tests (*χ* ^2^) were used to test for statistical significance with alpha (*p*) values <0.05 considered statistically significant. *Results.* Six of 10 findings (60%) were statistically significant. Statistically significant right-skew to rule out selective reporting was found (*χ* ^2^ = 38.8, *p* = 0.0001). Conversely, studies neither lacked evidential value (*χ* ^2^ = 6.8, *p* = 0.87) nor lacked evidential value and were intensely *p*-hacked (*χ* ^2^ = 4.3, *p* = 0.98). *Conclusion.* Evidential value results confirm that exercise reduces BMI *z*-score in overweight and obese children and adolescents, an important therapeutic strategy for treating and preventing CVD.

## 1. Introduction

Cardiovascular disease (CVD) is the leading cause of mortality from noncommunicable diseases worldwide, estimated in 2012 at 17.5 million, or 46.2%, of all noncommunicable disease deaths [1]. By 2030, it is estimated that mortality from CVD will increase to 23.3 million [2]. Two of the major risk factors for CVD are overweight and obesity [3, 4], a global and increasing problem among children and adolescents in both developed and developing countries. To illustrate, between 1980 and 2013, the worldwide prevalence of overweight and obesity among children and adolescents in developed countries increased from 16.9% to 23.8% for boys and from 16.2% to 22.6% for girls [5]. For children and adolescents in developing countries, increases ranged from 8.1% to 12.9% for boys and 8.4% to 13.4% for girls [5]. The deleterious effects of overweight and obesity during the childhood and adolescent periods are both immediate and long term. For example, in a population-based sample of United States (US) children and adolescents 5 to 17 years of age, approximately 70% of obese youth had at least one CVD risk factor [6]. From a long-term perspective, overweight and obesity during childhood and adolescence have been shown to track into adulthood [7], thereby placing this population at an increased risk for CVD and the mortality associated with such [3, 4]. The tracking of obesity into adulthood is especially noteworthy given that up to 80% of obese adolescents are at risk of becoming obese adults [8].

Therapeutic strategies aimed at reducing the prevalence of overweight and obesity among children and adolescents are important for reducing the lifetime risk of CVD. One such strategy is exercise, a low-cost, nonpharmacological approach that is available to the vast majority of overweight and obese children and adolescents. In a recent meta-analysis that included 835 overweight and obese children and adolescents, a statistically significant, exercise-induced reduction in BMI *z*-score was reported [9]. While these results are encouraging, all of the included studies were published in journals. This is problematic because studies published in journals suffer from an excess of statistically significant findings [10]. Consequently, such findings may not represent the true truth. This excess in statistically significant findings has been shown to be the result of factors that include, but are not necessarily limited to, selective reporting by investigators [11–17]. At all levels of use (research, practice, and policy), it is critically important to understand the true effects of exercise in overweight and obese children and adolescents. However, while guidelines for the assessment of selective reporting and related biases in meta-analysis exist, all have notable limitations and no adjustment methods are recommended [18]. Recently, however, *p*-curve, a new and novel method that can rule out selective reporting and does not require access to nonsignificant findings, has been developed and validated [19, 20]. Thus, given the CVD-related importance of understanding the true effects of exercise on adiposity in overweight and obese children and adolescents, the purpose of this study was to examine whether there is evidential value that exercise improves BMI *z*-score in overweight and obese children and adolescents.

## 2. Methods

### 2.1. Data Source

Data for the current study were derived from a recently published aggregate data meta-analysis that has previously been described in detail elsewhere [9]. Briefly, studies were included if they were randomized controlled trials examining the effects of exercise (aerobic, strength training, or both) on BMI *z*-score in overweight and obese children and adolescents [9]. A total of 10 studies representing 835 overweight and obese children and adolescents (456 exercise, 379 control) were included [21–30]. BMI *z*-score was chosen as the primary outcome based on previous research suggesting its greater validity over other types of BMI-related measures [31]. The focus was on a BMI-related measure over other measures of adiposity, for example, fat mass, given that BMI-related measures not only are the most common method for assessing adiposity, but also are used to define overweight and obesity in children, adolescents, and adults. BMI *z*-scores from each study were calculated by subtracting the change outcome difference in the exercise group from the change outcome difference in the control group and weighting by the inverse of the pooled variance. Overall results for BMI *z*-score from each included study were pooled using a random-effects model that incorporates heterogeneity into the analysis. Heterogeneity was assessed using Cochran's *Q* statistic and *I* ^2^ [32–34].

### 2.2. Determination of Evidential Value

To determine whether evidential value exists with respect to exercise improving BMI *z*-score in overweight and obese children and adolescents, *p*-curve, a recent and novel approach, was used [19, 20]. The objective of *p*-curve is to test for evidential value in order to eliminate selective reporting as a reason for statistically significant findings. Statistical inference includes (1) studies that contain evidential value (right-skew), (2) studies that lack evidential value (flatter than 33% power), and (3) studies that lack evidential value and were intensely *p*-hacked (left skew). It consists of the distribution of statistically significant *p* values <0.05 for a group of studies, with nonsignificant *p* values >0.05 not included in the analysis. Right-skewed *p* values are indicative of true effects and thus evidential value because they include a greater number of low (*p*s = 0.01) versus high (*p*s = 0.04) statistically significant alpha values. Probability values that are not right-skewed suggest a lack of evidential value while those that are left-skewed are suggestive of *p*-hacking, that is, investigator-suppression of subsets of nonsignificant results.

Testing for evidential value consisted of two steps. First, for each statistically significant *p* value <0.05, the probability of observing a significant *p* value at least as extreme as if the null were true was calculated. This is known as the *pp* value (*p* value of the *p* value) and was calculated by dividing statistically significant probability values from each study by 0.05. For this study, the probability values were derived from *z*-values calculated from the exercise minus control group differences in BMI *z*-score for each study. In order to maintain independence, the results from one study that included more than one intervention group were collapsed so that only one probability value was included for that study [22]. The second step consisted of pooling the *pp* values using Fisher's method [35]. This yields an overall *χ* ^2^ test for skew with degrees of freedom equal to twice the number of *p* values. Thus, a statistically significant *χ* ^2^ test is indicative of a significant right-skewed *p*-curve and thus evidential value that exercise improves BMI *z*-score in overweight and obese children and adolescents. The absence of a statistically significant right-skewed *p* value suggests either a lack of information to make inferences about evidential value or a lack of evidential value. To test for a lack of information, that is, power, the same approach as for right-skew was used except that *pp* values were recalculated for expected *p*-curves using a power of 33% and the study's sample size, accomplished via the use of noncentral distributions. To test for a lack of evidential value suggestive of intense *p*-hacking (left skew), the same approach was used as for testing for evidential value of a real effect, that is, right-skew, except that the *pp* values for left skew were calculated as 1 minus the right-skew *pp* value. All calculations were robust to outliers, with *pp* values winsorized at 0.01 and 0.99. Chi-squared probability values ≤0.05 were considered statistically significant. Data were analyzed using *p*-curve (version 2.0), a free online statistical program available at http://www.p-curve.com/app2/, version 3.0 of Comprehensive Meta-Analysis [36], and Microsoft Excel 2010 [37].

## 3. Results

### 3.1. Changes in BMI *z*-Score

**Figure 1:**
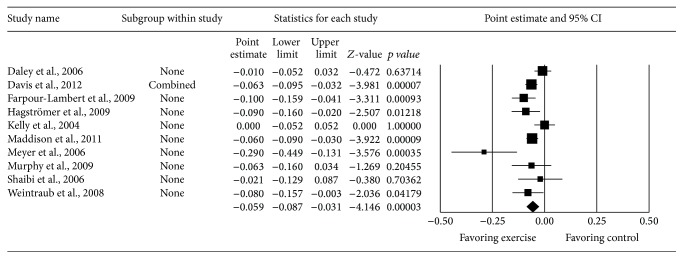
Forest plot for study-level changes in BMI *z*-score. The black squares represent the mean difference while the left and right extremes of the squares represent the corresponding 95% confidence intervals. The middle of the black diamond represents the overall mean difference while the left and right extremes of the diamond represent the corresponding 95%confidence intervals.

Figure 1 shows a forest plot that depicts the overall results for changes in BMI *z*-score, details of which have been previously described [9]. As can be seen, a statistically significant reduction in BMI *z*-score in favor of exercise was observed as well as nonoverlapping 95% confidence intervals. Heterogeneity was statistically significant (*Q* = 21.5, *p* = 0.01) but moderate (*I* ^2^ = 58.2%, 95% confidence interval = 15.7% to 79.2%). Changes in BMI *z*-score ranged from −0.29 to 0 while overall results were equivalent to a relative reduction of approximately 2%. Six of 10 results (60%) were statistically significant (*p* < 0.05).

### 3.2. *p*-Curve Results

**Figure 2:**
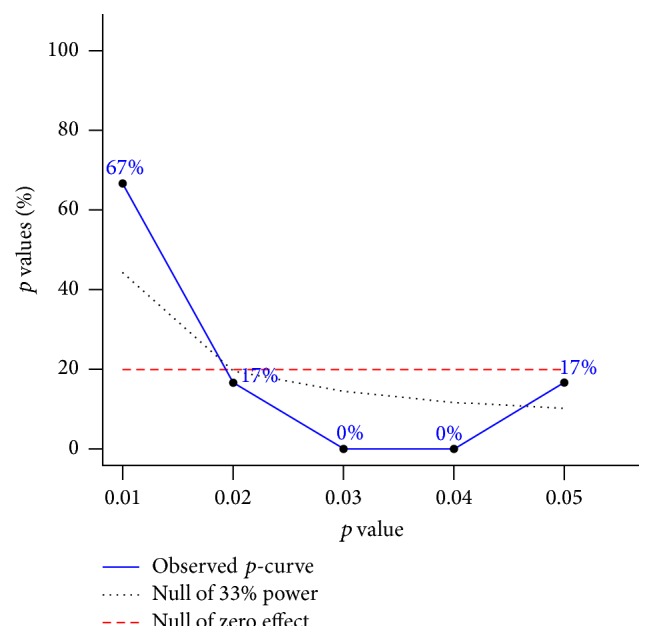
*p*-curve results. *p*-curve results for evidential value. Results are significantly right-skewed (*p* = 0.001), suggesting that evidential value exists that exercise improves BMI *z*-score in overweight and obese children and adolescents. The graphed results include six statistically significant *p* values <0.05. Four additional results were entered but excluded from the analysis because of nonsignificance (*p* ≥ 0.05). All graphed calculations were adjusted for outliers by winsorizing *pp* values at 0.01 and 0.99.

Results for evidential value are shown in Table 1 and Figure 2. As can be seen, there was statistically significant right-skew and thus evidential value that exercise reduces BMI *z*-score in overweight and obese children and adolescents. Consistent with this finding are the nonsignificant results for power and left skew.

## 4. Discussion

### 4.1. Overall Findings

The purpose of this study was to determine whether there is evidential value that exercise improves BMI *z*-score in overweight and obese children and adolescents. The findings indicate that the included studies contain evidential value that exercise improves BMI *z*-score in overweight and obese children and adolescents and provide much-needed reinforcement to previous work on this topic [9]. These results are important given that (1) overweight and obesity are two of the major risk factors for CVD [3, 4], (2) the worldwide prevalence of overweight and obesity in children and adolescents is high [5], and (3) a need exists to develop therapeutic strategies aimed at reducing CVD. The former notwithstanding, the results could be questioned given that the magnitude of improvement in BMI *z*-score was approximately 2% and *p*-curve does not directly assess for such. However, as previously reported, gross estimates suggest that approximately one million overweight and obese children and adolescents worldwide could reduce their BMI *z*-score by exercising regularly [9]. From the investigators' perspective, these findings are important.

### 4.2. Implications for Research and Practice

The results of the current study provide overall results in relation to the effects of exercise on BMI *z*-score in overweight and obese children and adolescents. However, the dose-response effects of exercise were not examined and when previously examined [9] did not glean any substantive findings. Given the former and as previously suggested [9], a need exists to examine the dose-response effects of exercise in a representative sample of this population. Until that time, adherence to the World Health Organization (WHO) recommendation of 60 minutes per day of physical activity for children and adolescents appears appropriate and is in concordance with the WHO year 2025 goals of reducing the global prevalence of physical inactivity across all age groups by 10% as well as halting the rise in obesity [38]. Increased participation in exercise among overweight and obese children and adolescents will also likely contribute to the WHO year 2025 goal of reducing mortality from cardiovascular diseases, cancer, diabetes, or chronic respiratory diseases by 25% [38].

### 4.3. Strengths and Potential Limitations

The major strength of the current study is the use of a recent and novel approach to address selective reporting of results [19, 20] and thus provide more convincing evidence regarding the true effects of exercise on BMI *z*-score in overweight and obese children and adolescents. This is critically important given the prevalence of self-report bias and subsequent overestimation of treatment effects in the published literature [11–17]. In contrast, one potential limitation is the fact that the current findings were based on 835 overweight and obese children and adolescents nested within 10 studies [21–30]. Consequently, there may have been a lack of precision given that the larger the number of studies as well as number of subjects nested within each study, the greater the precision of *p*-curve results [19]. Another potential limitation is that *p*-curve can fail to detect studies that lack evidential value because it is significantly right-skewed [20]. Furthermore, *p*-curve does not include *p* values >0.05, including those close to 0.05. As a result, *p* values suggestive of no effect, while extremely infrequent in the presence of a genuine effect, are excluded [20].

## 5. Conclusions

The results of the current study provide evidential value that exercise reduces BMI *z*-score in overweight and obese children and adolescents. Given its CVD-related importance, exercise should be recommended as a therapeutic strategy for reducing BMI *z*-score in overweight and obese children adolescents.

## Figures and Tables

**Table 1:** Evidential values for changes in BMI *z*-score.

Statistical inference	*χ* ^2^	df	*p*
Studies contain evidential value (right-skewed)	38.8	12	0.0001^*∗*^
Studies lack evidential value (flatter than 33% power)	6.82	12	0.87
Studies lack evidential value and were intensely *p*-hacked (left-skewed)	4.27	12	0.98

Notes: *χ* ^2^, chi-squared tests; df, degrees of freedom (2 × the number of statistically significant values); *p*, probability value; ^*∗*^statistically significant (*p* < 0.05); calculations robust to outliers with *pp* values winsorized at 0.01 and 0.99.
